# Preparation of Polydimethylsiloxane-Modified Waterborne Polyurethane Coatings for Marine Applications

**DOI:** 10.3390/polym13244283

**Published:** 2021-12-07

**Authors:** Min-Gu Kim, Kyoung-Il Jo, Eunji Kim, Jae-Hyung Park, Jae-Wang Ko, Jin Hong Lee

**Affiliations:** 1 Industry Fiber Laboratory, Korea Institute of Footwear & Leather Technology (KIFLT), Busan 47454, Korea; kimmingu95@pusan.ac.kr (M.-G.K.); kyeoungil@gmail.com (K.-I.J.); jhpark@kiflt.re.kr (J.-H.P.); 2 School of Chemical Engineering, Pusan National University, Busan 46241, Korea; eunjikim@pusan.ac.kr

**Keywords:** waterborne polyurethanes, polyurethane coatings, polydimethylsiloxanes, hydrophobicity, marine applications

## Abstract

A series of waterborne polyurethane dispersions (WPUs) modified with hydroxyl-terminated polydimethylsiloxane (PDMS) were prepared by incorporating PDMS into the soft segments of polyurethane chains. The structural characteristics of the prepared samples were analyzed by Fourier transform infrared spectroscopy (FTIR), X-ray diffraction (XRD), X-ray photoelectron spectroscopy (XPS), and particle size analysis (PSA). The effect of PDMS content on the thermal and mechanical properties of PDMS-modified waterborne polyurethanes (PS-WPU) was investigated. In addition, the water resistance and dimensional stability of the PS-WPU were investigated by measuring its water absorption ratio and water contact angle along with universal testing machine measurements.

## 1. Introduction

Polyurethane (PU), a polymer joined by urethane bonds, is produced by the reaction of polyols with isocyanates. Currently, PUs are one of the most commonly used materials in various applications such as coatings [1,2], adhesives [3,4], foams [5,6], and elastomers [7,8]. The properties of PU are dependent on the type of polyols and isocyanates used, and the soft segment of PU is formed from a polyol, while the hard segment is composed of an isocyanate and a chain extender.

In recent years, with increasingly strict regulations for volatile organic compounds, waterborne polyurethanes (WPUs), which utilize water as a dispersion medium, have received significant attention [9]. WPUs are synthesized by introducing hydrophilic segments or ionic groups, which act as emulsifiers, into the molecular chains of the polymers [10]. WPUs have been applied in a wide range of adhesives and coatings for textile, plastic, metal, and offshore structures owing to their tunable properties, which can be controlled by the proportion of soft and hard segments [9,11]. However, their low resistance in water and solvents, apart from limited thermal stability and mechanical properties, needs to be improved in order to replace solvent-based PUs.

Considerable efforts have been devoted into overcoming these problems, including the introduction of a network structure enabled by a crosslinking reaction of epoxy [12] and acrylic groups [13,14], blending WPU with different polymers to form interpenetrating networks (IPNs) [15], modifying the chemical structures of WPUs with nanoparticles [16,17], and copolymerization [18,19]. Another promising approach is the use of polydimethylsiloxane (PDMS) because of its advantageous properties, such as high thermal stability, biocompatibility, flexibility, water resistance, and low surface energy [20,21,22,23,24,25]. Vlad et al. reported that IPNs prepared by the combination of castor-oil-based PUs and PDMS provide enhanced mechanical properties. However, the IPNs exhibited significant phase separation due to a large difference in the solubility parameter between the PU network and PDMS network [26]. Compared with solvent-based PUs, WPUs containing ionic moieties or hydrophilic segments are more polar; this results in a large polarity difference between WPUs and PDMS and their poor compatibility. Hence, research has largely focused on chemical modifications to increase the compatibility of WPUs and PDMS [27,28].

In this study, different amounts of hydroxyl-terminated PDMS were incorporated into the molecular chains of WPU as soft segments to investigate the effects of the PDMS content on the mechanical and thermal properties of PDMS-modified PU. Analytical results revealed that an increase in the PDMS content resulted in improved thermal stability, elongation properties, and water resistance, while decreasing the tensile strength. These findings are important for understanding the potential marine applications of PDMS-modified PUs.

## 2. Materials and Methods

### 2.1. Materials

Polytetramethylene ether glycol (PTMG, Aldrich, St. Louis, MO, USA) (M_n_ = 2000 g mol^−1^), hydroxyl-terminated PDMS (Aldrich) (M_n_ = 4200 g mol^−1^), isophorone diisocyanate (IPDI, Aldrich; 98%), 2,2-bis(hydroxymethyl) propionic acid (DMPA, Aldrich; 98%), dibutyltin dilaurate (DBTDL, Aldrich; 95%), triethylamine (TEA, Samchun Chemical Co., Ltd. Pingze, Korea; 99%), and ethylenediamine (EDA, Samchun Chemical Co., Ltd.; 99%) were used as received.

### 2.2. Preparation of PS-WPU

**Scheme 1:**
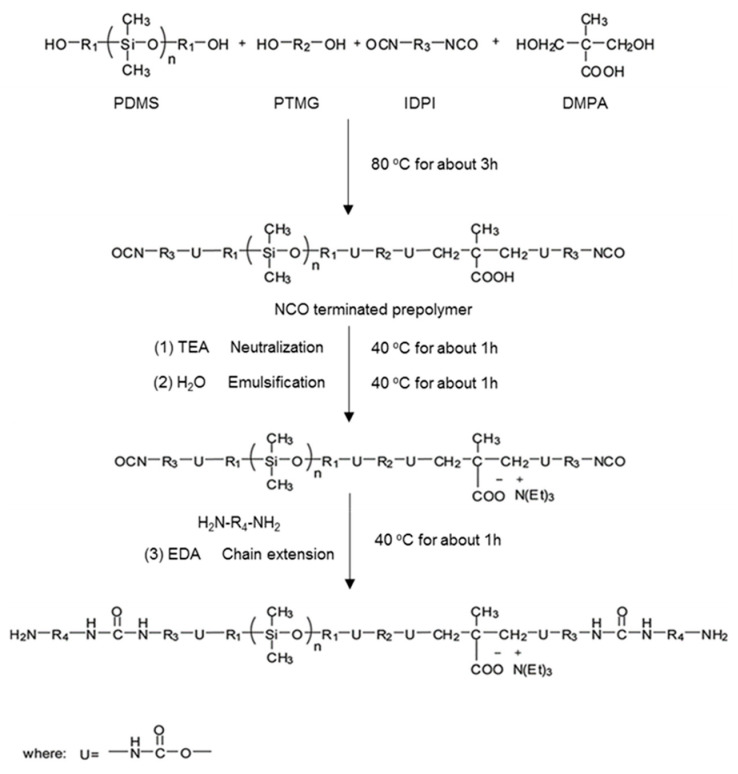
Preparation of PS-WPU.

A series of WPU samples with different PDMS contents (0, 5, 10, 15 and 20%) were synthesized using the prepolymer method as follows: PTMG, PDMS, and DMPA were first placed in a three-necked glass reactor equipped with a physical stirrer, dropping funnel, and a screw tube cooler. The mixture was then stirred at 80 °C. After 1 h, IPDI and DBTDL were added dropwise to the reactor. The reaction was carried out at 80 °C for 3 h to obtain the NCO-terminated prepolymer. During the reaction, acetone was added to adjust the viscosity of the solution. After reaction completion, the solution was cooled to 40 °C, and then TEA was added to neutralize the carboxyl groups of PU. After 1 h of neutralization, distilled water was added with vigorous stirring for 1 h, and then EDA was added for chain extension for another 1 h. Finally, acetone was removed under vacuum to obtain the WPU dispersion. The WPU films were prepared by casting the WPU dispersion into a glass mold and drying at room temperature until all the solvents had evaporated. The sample designations and compositions are summarized in Table 1, and the reaction process is illustrated in Scheme 1.

**Table 1:** Composition of PS-WPU samples.

Samples	Composition (mol)					
	PDMS	PTMG	DMPA	IPDI	TEA	EDA
PS-WPU 1	0	0.033	0.067	0.12	0.067	0.02
PS-WPU 2	0.00165	0.03135	0.067	0.12	0.067	0.02
PS-WPU 3	0.0033	0.0297	0.067	0.12	0.067	0.02
PS-WPU 4	0.00495	0.02805	0.067	0.12	0.067	0.02
PS-WPU 5	0.0066	0.0264	0.067	0.12	0.067	0.02

### 2.3. Characterization

Fourier-transform infrared (FTIR) spectra of the samples were analyzed using an FTIR spectrometer (JASCO Co., FT/IR-6200, Tokyo, Japan) using attenuated total reflection (ATR) method. The spectra were recorded in the range of 4000–400 cm^−1^ at a resolution of 4 cm^−1^, and each sample was scanned 64 times.

The particle sizes of the samples were determined using a particle size analyzer (PSA) (Malvern Panalytical, Mastersizer 3000, Malvern, UK). The samples were diluted to a concentration of 0.01% using deionized water (D.W).

X-ray diffraction (XRD) patterns were obtained using an X-ray diffractometer (Philips, X’pert 3, Eindhoven, Netherlands) with Cu-Kα radiation (λ = 1.54060 Å) at 40 kV and 30 mA. The angle was between 20° and 40° with a step size of 0.01°.

Qualitative and quantitative information on the surface elemental compositions of the samples were obtained via X-ray photoelectron spectroscopy (XPS, Thermo Fisher Scientific, Waltham, MA, USA) with Al K*a* achromatic X-ray. The spectra were recorded in the range of 0–1300 eV under 90° take-off angles.

Dynamic mechanical analysis (DMA) was conducted using a DMA instrument (TA Instruments, DMA 850, New Castle, DE, USA). The temperature was in the range of −100 °C to 100 °C, with a heating rate of 5 °C/min at an oscillating frequency of 1 Hz.

Thermogravimetric analysis (TGA) and derivative thermogravimetric (DTG) analysis were conducted using a thermal analyzer (TA Instruments, TGA Q500, New Castle, DE, USA) at a heating rate of 10 °C/min in a nitrogen atmosphere, with temperatures in the range of 100 °C to 800 °C.

The tensile properties were measured using a universal testing machine (Instron Co., Instron 3345, Canton, OH, USA) at a crosshead speed of 50 mm/min at room temperature and −60 °C. The mechanical test on each dumbbell-shaped sample was conducted five times, and the average value was calculated according to the ASTM D638.

Water contact angles (WCAs) were measured with a contact angle goniometer (Femtobiomed, Smart Drop Standard, Seongnam, Korea) at room temperature with water droplets (deionized water (D.W) and seawater (S.W)). Each sample was analyzed thrice at three different locations, and the average value of the contact angle was calculated. The surface free energy was calculated by inputting the value of the contact angle to the surface energy calculation software.

To measure the swelling in water, the films were immersed in water (D.W and S.W) at 25 °C. The water swelling properties were calculated from the differences in the mass of the samples using the following equation:

$$Swelling\;ratio=\frac{100\left(W-W_{0}\right)}{W_{0}}$$

where *W*_0_ and *W* represent the initial and final (after water swelling) film weights, respectively.

## 3. Results and Discussion

### 3.1. FTIR Analysis

**Figure 1:**
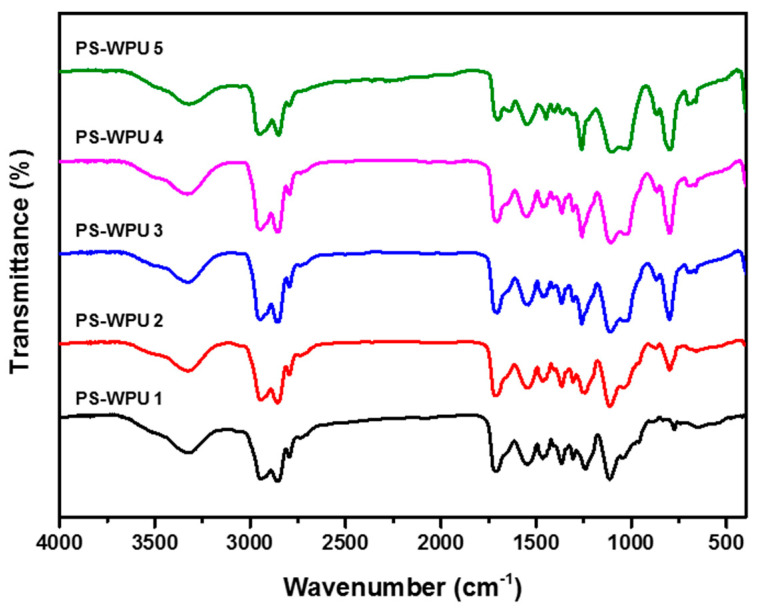
FTIR spectra of PS−WPU samples.

The chemical structures of the PS-WPU samples are investigated by FTIR spectroscopy, as shown in Figure 1. All the samples indicate the absorption peaks of typical PU at 1530 cm^−1^ (–NH bending in urethane), 1717 cm^−1^ (–C=O stretching in urethane), 1160 cm^−1^ (C–O–C ether group) and 2923–2850 cm^−1^ (C–H symmetric and asymmetric stretching of CH_2_ group, respectively) [29]. The absorption peaks near 3480 cm^−1^ and 3320 cm^−1^ indicate free NH stretching and hydrogen-bonded NH stretching, respectively [30]. The absence of an NCO peak at 2270 cm^−1^ in all the samples indicates the complete reaction of the NCO group. Furthermore, except for PS-WPU 1, all the remaining PS-WPU samples indicate the characteristic absorption peaks of Si–CH_3_ at 1261 cm^−1^ (Si–CH_3_ deformation), Si–O–Si at 1023 cm^−1^ (Si–O stretching), and Si–CH_3_ at 806^−10^ (CH_3_ rocking and Si–C stretching), which indicates that PDMS was successfully incorporated into WPU [31]. In addition, the intensity of the absorption peak increases with increasing PDMS content.

### 3.2. Particle Size of the PS−WPU Dispersions

**Figure 2:**
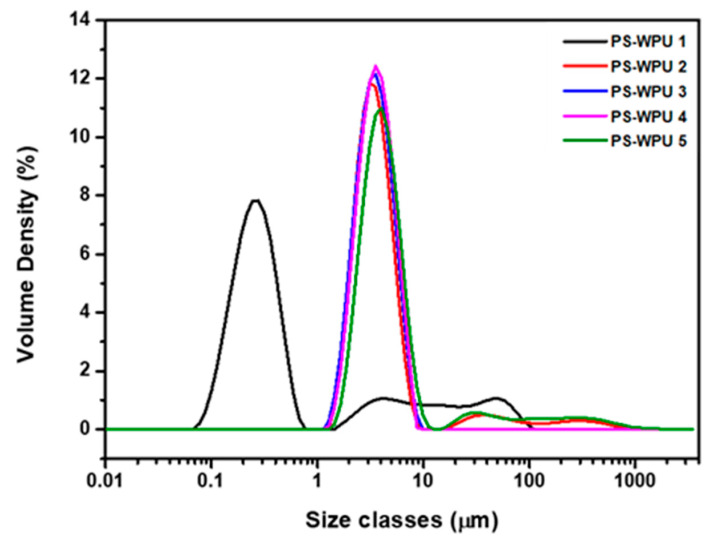
Particle size distribution of PS-WPU samples.

Figure 2 and Table 2 present the particle size distribution of the PS-WPU dispersions with different PDMS contents. The average particle size of the PS-WPU dispersions increase with increasing PDMS content from 0.331 to 4.45 μm. However, the PS-WPU dispersions exhibited bimodal particle-size distributions. These results may be ascribed to the hydrophobicity of the PDMS chains. During the formation of colloidal particles in the water-based dispersions, the hydrophilic groups and segments tend to be on the outer layers, while the hydrophobic PDMS favored the inner layers. As a result, a higher content of PDMS results in a larger free volume and lower chain packing density in the interior of the colloidal particles, and thus, larger particles [1,32,33,34]. Furthermore, all the PS-WPU dispersions showed good stability with no precipitation at room temperature for a period of three months.

### 3.3. XRD and XPS Analyses

**Figure 3:**
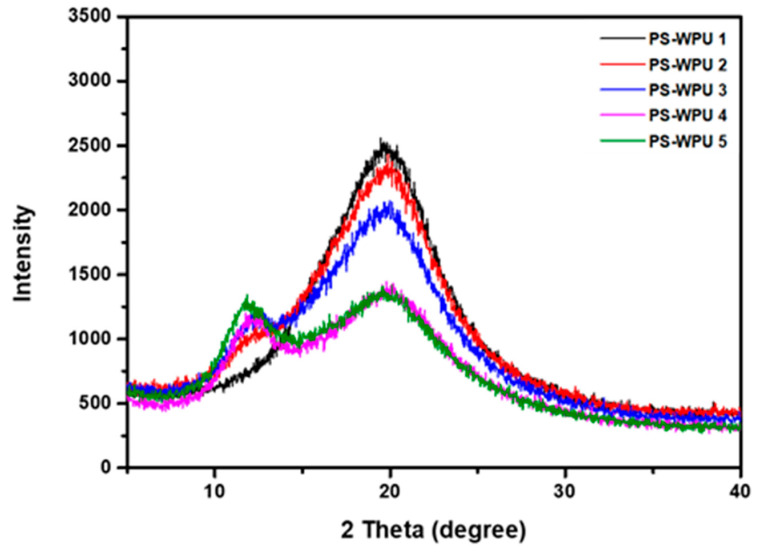
X-ray diffraction patterns of PS-WPU samples.

The crystalline structures of the PS-WPUs were investigated using XRD. As shown in Figure 3, there are no sharp peaks in the diffraction patterns, which indicates the amorphous structures of the PS-WPUs. All the PS-WPU samples show a broad peak centered at 2θ = 20°, indicating the typical crystalline nature of PU. The crystallinity of PU is due to the hydrogen bonds that occur in the molecules [35,36]. PDMS exhibits a characteristic amorphous halo at 2θ of 12° [37]. All of the samples, except that of PS-WPU 1, show characteristic peaks corresponding to PDMS, and the peak intensity increases with increasing PDMS content. Furthermore, the diffraction pattern becomes weaker and broader, which indicates that the crystallinity and orientation of the PU chains are disturbed by the addition of PDMS.

**Figure 4:**
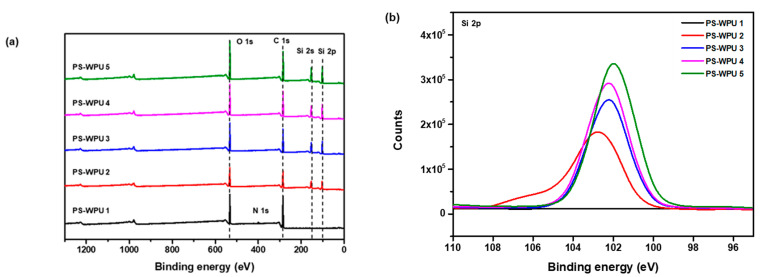
(**a**) XPS spectra and (**b**) Si 2p XPS spectra of the PS-WPU samples.

The elemental composition of the PS-PU coating surface was investigated using XPS. Figure 4a shows the XPS spectra of PS-WPU 1 observed at binding energies of 284, 399, and 532 eV, which are attributed to C 1s, N 1s, and O 1s, respectively. The PDMS-modified samples (PS-WPU 2, 3, 4, and 5) exhibit strong characteristic Si 2p and Si 2s peaks at 102 and 155 eV, respectively. In addition, as the PDMS content increases, the Si atomic content increases from 0 to 20.85% (Figure 4b and Table 3). The presence of the Si peak and the increase in the atomic concentration of Si atoms indicate the successful incorporation of PDMS, which further confirm the FTIR and XRD results.

### 3.4. DMA Analysis

**Figure 5:**
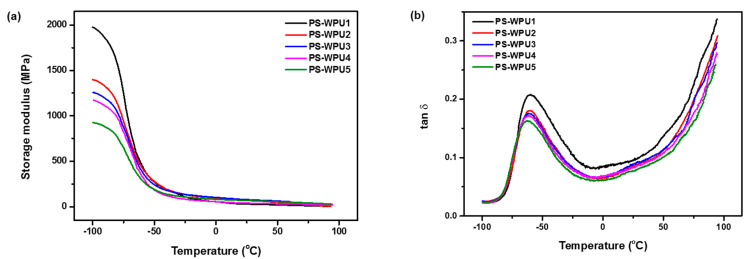
Temperature dependence of (**a**) storage modulus and (**b**) tan δ of PS-WPU samples.

The viscoelastic properties of the PS-WPU samples were investigated using DMA. Figure 5 presents the storage modulus and damping factor (tan δ) of the PS-WPUs as a function of temperature. As shown in Figure 5a, the storage modulus curves of all PS-WPU samples decrease abruptly over the temperature range from −100 °C to −35 °C, which is ascribed to the glass transition in the soft segment [38]. PS-WPU 1 without PDMS exhibits the highest storage modulus, and the storage modulus of the PS-WPU samples decreases with increasing PDMS content. The storage modulus is a measure of material stiffness [39,40,41], and the incorporation of PDMS into the soft segment of the WPU decreases the stiffness because of the high flexibility of the PDMS. Figure 5b displays the tan δ curves, which are directly related to the T_g_ of the PS-WPU samples. The relatively sharp peak in the tan δ curves is ascribed to the primary dispersion (α_a_) associated with the glass transition of PU. In this transition temperature region, the storage modulus decreases abruptly, and the micro-Brownian motions of the PU chains become clear in the soft domains [42]. The values of T_g_ decrease from −59.87 °C to −62.49 °C with increasing PDMS content because of the enhanced motion of PDMS chains.

### 3.5. TGA and DTG Analyses

**Figure 6:**
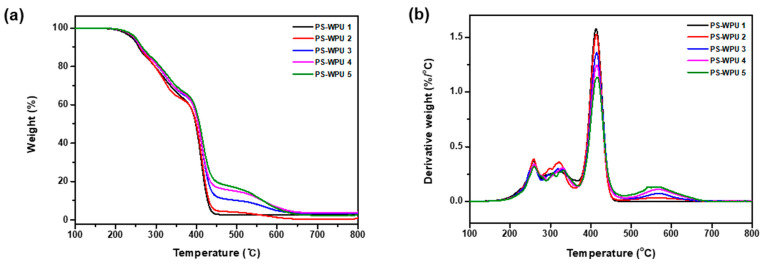
(**a**) TGA and (**b**) DTG curves of PS-WPU samples.

To investigate the effect of PDMS on the thermal stability, TGA and DTG analyses were carried out. The results are shown in Figure 6. The characteristic degradation temperatures associated with the thermal degradation are summarized in Table 4. All the PS-WPU samples exhibit similar thermal decomposition behaviors. The slight weight loss before 200 °C is caused by the volatilization of solvents in the PS-WPU film. The temperature at which 10% weight loss occurs (T_10%_) and the mid-point temperature of the weight loss (T_50%_) are employed to determine the thermal stability of the PS-WPU films, as shown in Table 4. Both the weight loss temperatures, T_10%_ and T_50%_, increase with increasing PDMS content. The improvement in thermal stability indicates that PDMS plays an important role in preventing the decomposition of the main chain of WPU. This improved stability can be attributed to the higher energy of the Si–O bond (~460 kJ/mol) compared to the energies of the C–C and C–O bonds (~345 kJ/mol) [43,44].

According to the TGA and DTG curves, all the PS-WPU samples exhibit a three-step degradation profile. The first degradation step from 250 °C to 350 °C is related to the decomposition of the hard segment of PU, since the C–N bond breaks more easily than the C–O and C–C bonds [45,46]. The second degradation step at around 400 °C is due to the scission of the soft segment. The third degradation step above 500 °C may correspond to the depolymerization of residual components [47]. These results indicate that increasing the PDMS content improves the thermal stability of the PS-WPU samples.

**Table 4:** Thermal properties of PS-WPU samples.

Samples	Decomposition Temperature		
	T_10%_ (°C)	T_50%_ (°C)	T_max%_ (°C)
PS-WPU 1	257.4	398.3	412.7
PS-WPU 2	260.5	399.9	413.7
PS-WPU 3	262.3	404.4	414.8
PS-WPU 4	262.4	404.5	415.7
PS-WPU 5	262.4	407.9	416.8

### 3.6. Mechanical Properties

**Figure 7:**
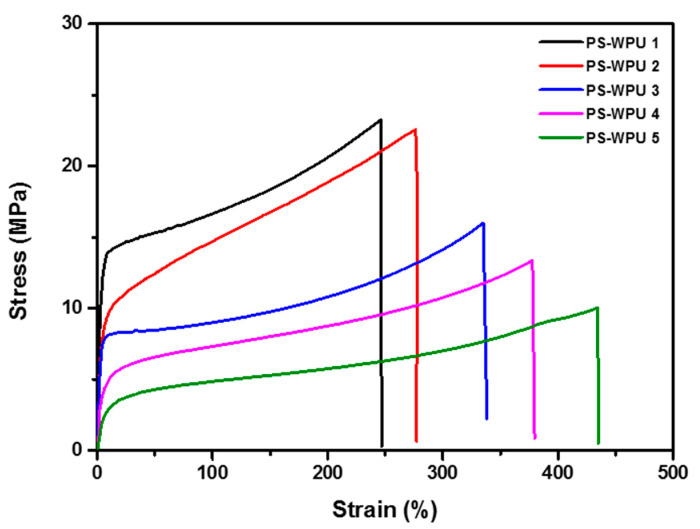
Stress–strain curves of PS-WPU samples obtained at room temperature.

The stress–strain curves of the PS-WPU samples are shown in Figure 7, and the obtained values of tensile strength, elongation at break, and modulus are summarized in Table 5. The PS-WPU 1 without PDMS shows a maximum stress of 23.27 MPa, modulus of 4.42 MPa, and strain of 246% at room temperature. Tensile strength and modulus gradually decrease with increasing PDMS content from 23.27 to 10.05 MPa and 4.42 to 0.51 MPa, respectively. However, the strain at break increases from 246 to 434% with increasing PDMS content. As shown in Table 5, a similar trend is observed at −60 °C. With increasing PDMS content, tensile strength and modulus decrease from 32.84 to 20.97 MPa and 11.81 to 3.59 MPa, respectively. The elongation at break increases from 211 to 392%. Interestingly, the obtained tensile strength and modulus values at −60 °C are higher than those obtained at room temperature. The elongation at break is lower at −60 °C than at room temperature. These results indicate that the incorporation of PDMS can improve the flexibility, elasticity, and low temperature properties [31].

### 3.7. Surface Hydrophobicity and Water Resistance

**Figure 8:**
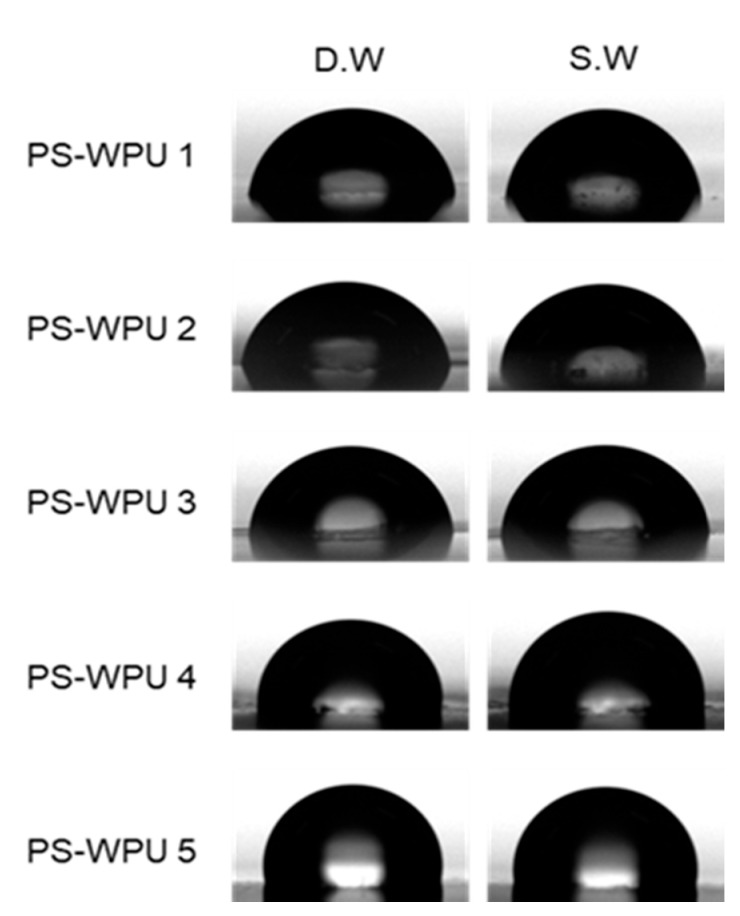
Water contact angles images of the recorded droplets (D.W and S.W) on the surface of PS-WPUs.

The applicability of polymer coating materials can be predicted based on hydrophobicity and water resistance in different testing media. In this study, the hydrophobicity and swelling behavior were investigated using D.W and S.W (5% NaCl). The surface hydrophobicity of the PS-WPU films is investigated by measuring the WCA between the water droplet D.W and S.W) and the surface of the film, as shown in Figure 8. The surface energy (Table 6) was also calculated from the contact angle values using the Owens equation [48]:

$$\gamma_{SV}=\gamma_{SV}^{d}+\gamma_{SV}^{p}$$

$$\gamma_{LV}\left(1+cos\theta_{LV}\right)=2{\left(\gamma_{LV}^{d}\gamma_{SV}^{d}\right)}^{1/2}+2{\left(\gamma_{LV}^{p}\gamma_{SV}^{p}\right)}^{1/2}$$

where *γ_LV_* is the surface tension of liquid, *θ* is the contact angle of liquid on the surface, $\gamma_{LV}^{d}$ and $\gamma_{LV}^{p}$ are the dispersive and polar components of the liquids, respectively, and $\gamma_{SV}^{d}$ and $\gamma_{SV}^{p}$ are the dispersive and polar components of the solids, respectively. The dispersion and polar components of water are 21.8 and 51.0 mN/m, respectively. The dispersion and polar components of formamide are 39.0 and 19.0 mN/m, respectively.

The WCAs of the PS-WPU 1 film without PDMS for D.W and S.W are 80.3° and 82.0°, respectively. With increasing PDMS content from 0% to 20%, the WCA for D.W and S.W increases from 80.3° to 101.8° and 82.0° to 99.5°, respectively. In addition, the surface energy of the PS-WPU films decreases with increasing PDMS content for D.W and S.W. For D.W, the surface energy of the PS-WPU films decreases from 34.1 mN/m to 20.9 mN/m with increasing PDMS content. For S.W, the surface energy of the PS-WPU films decreases from 46.6 mN/m to 34.1 mN/m with increasing PDMS content. This result confirms that the incorporation of PDMS can improve the surface hydrophobicity of the copolymers. The presence of siloxane provides a thermodynamic driving force for siloxane segments to migrate toward the air–polymer interface of the film during film formation. As a result, a siloxane-enriched surface is formed, which has a low surface energy because of weak intermolecular forces between the methyl groups (–CH_3_) and the strong (Si–O) and flexible (Si–O–Si) siloxane chain [49,50].

**Figure 9:**
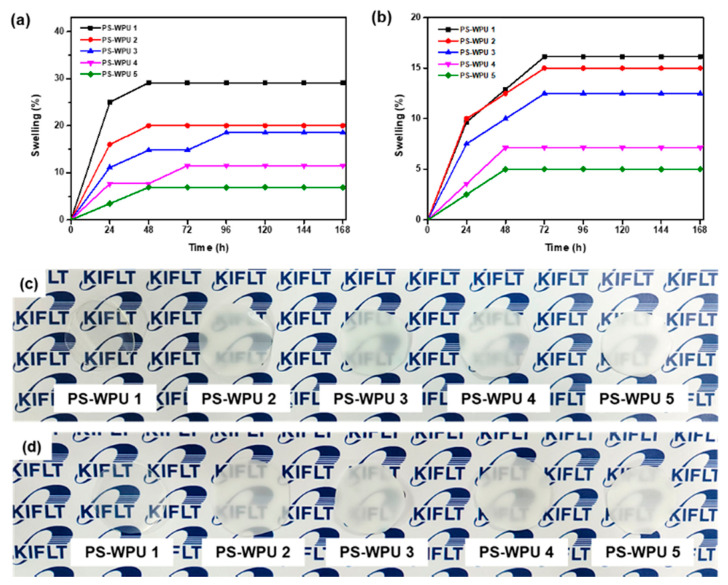
Water swelling ratio of the PS-WPU samples in (**a**) D.W and (**b**) S.W Images of the PS-WPU samples (**c**) before and (**d**) after swelling in D.W.

Water resistance is an important factor in coating materials. The water resistance of polymers is an attribute of their hydrophobicity, as well as the interaction between macromolecules [51,52]. To evaluate the water resistance of the PS-WPU films, the samples were immersed in water (D.W and S.W) for 168 h. The relationship between the water swelling ratio and soaking time of the PS-WPU films is shown in Figure 9a,b. The swelling ratio increases with increased soaking time up to 72 h and then levels off. The water swelling ratio of the PS-WPU films decreases with increasing PDMS content. The improved water resistance may be ascribed to the hydrophobicity of PDMS. When the films are immersed in water, the water fills the microcavities and they swell due to the interaction between the hydrophilic groups and water, resulting in the absorption of water. However, incorporating hydrophobic PDMS creates a hydrophobic barrier on the surface to prevent water absorption and reduce the interaction between hydrophilic groups and water [31,41,53,54]. Thus, it can be concluded that by incorporating hydrophobic PDMS into the soft segment of the PU chains, the water resistance of the PS-WPU films was enhanced. These results suggest that PS-WPUs have potential as coating materials for S.W repellent applications.

**Table 6:** Performance of the PS-WPU samples.

Samples	Water Contact Angle (°)		Surface Energy (γ) (mN/m)		Maximum Water Absorption (%)	
	D.W	S.W	D.W	S.W	D.W	S.W
PS-WPU 1	80.3	82.0	34.1	46.6	29.2	16.1
PS-WPU 2	83.6	83.9	32.0	45.2	20.0	15.0
PS-WPU 3	85.1	85.6	31.1	44.0	18.5	12.5
PS-WPU 4	95.0	95.0	25.1	37.3	11.5	7.1
PS-WPU 5	101.8	99.5	20.9	34.1	6.9	5.0

## 4. Conclusions

In this study, a series of PDMS-modified WPUs were synthesized with various PDMS contents. FTIR and XPS analyses confirmed the successful incorporation of PDMS into the PU soft segment. The average particle size of the PS-WPU samples increased with increasing PDMS content because of the large free volume and low chain packing density in the interior of the colloidal particles. XRD analysis showed that all the PS-WPU samples were amorphous, and the orientation of the PU chains was disturbed by the PDMS content. DMA results showed that the storage modulus and T_g_ of the samples decreased with increasing PDMS content. According to the TGA and DTG results, the incorporation of PDMS increased the thermal stability because of the higher energy of Si–O bonds compared to the C–C and C–O bonds. The tensile strength decreased, but the elongation at break increased with increasing PDMS content at 25 °C and −60 °C owing to the flexibility, poor tensile strength, and excellent low temperature properties of PDMS. In addition, the WCA and water resistance results confirmed that both D.W and S.W had good surface hydrophobicity and water resistance. According to all the results, the PS-WPU samples in this study represent excellent candidates for potential use as coatings in marine applications.

## Figures and Tables

**Table 2:** Particle size of PS-WPU samples.

Samples	Particle Size (μm)
PS-WPU 1	0.331
PS-WPU 2	3.65
PS-WPU 3	3.70
PS-WPU 4	3.79
PS-WPU 5	4.45

**Table 3:** Atomic relative concentration of elements in PS-WPU samples.

Samples	O 1s	N 1s	C 1s	Si 2p
PS-WPU 1	45.02	1.82	53.16	0
PS-WPU 2	45.87	0.79	35.57	17.77
PS-WPU 3	46.42	0.62	34.85	18.11
PS-WPU 4	46.82	0.34	33.26	19.58
PS-WPU 5	46.39	0.30	32.46	20.85

**Table 5:** Mechanical properties of PS-WPU samples.

Samples	Tensile Strength (MPa)		Elongation at Break (%)		Young’s Modulus (MPa)	
	25 °C	−60 °C	25 °C	−60 °C	25 °C	−60 °C
PS-WPU 1	23.27	32.84	246	211	4.42	11.81
PS-WPU 2	17.94	28.18	277	223	2.58	7.12
PS-WPU 3	16.01	25.56	335	298	1.89	6.04
PS-WPU 4	13.37	22.84	377	325	1.56	5.82
PS-WPU 5	10.05	20.97	434	392	0.51	3.59

## Data Availability

The data presented in this study are available on request from the corresponding author.
